# Supplementary material for: Use of oral polio vaccine and the incidence of COVID-19 in the world
Source: PLoS One. 2022 Mar 17;17(3):e0265562. doi: 10.1371/journal.pone.0265562 (PMC8929581; doi:10.1371/journal.pone.0265562)
Supplement: Attachment — Submitted filename: Answers to Reviewers Comments.docx [file pone.0265562.s002.docx]

**View Letter**

| **Date:** | Dec 01 2021 12:39AM |
| --- | --- |
| **To:** | "Robert C. Gallo" rgallo@ihv.umaryland.edu |
| **From:** | "PLOS ONE" plosone@plos.org |
| **Subject:** | PLOS ONE Decision: Revision required [PONE-D-21-33498] |

PONE-D-21-33498

Use of Oral Polio Vaccine and the Incidence of COVID-19 in the World

PLOS ONE

Dear Dr. Robert C. Gallo,

Thank you for submitting your manuscript to PLOS ONE. After careful consideration, we feel that it has merit but does not fully meet PLOS ONE’s publication criteria as it currently stands. Therefore, we invite you to submit a revised version of the manuscript that addresses the points raised during the review process.

Please submit your revised manuscript by Dec 31, 2021. If you will need more time than this to complete your revisions, please reply to this message or contact the journal office at [plosone@plos.org](mailto:plosone@plos.org). When you're ready to submit your revision, log on to https://www.editorialmanager.com/pone/ and select the 'Submissions Needing Revision' folder to locate your manuscript file.

Please include the following items when submitting your revised manuscript:

- A rebuttal letter that responds to each point raised by the academic editor and reviewer(s). You should upload this letter as a separate file labeled 'Response to Reviewers'.
- A marked-up copy of your manuscript that highlights changes made to the original version. You should upload this as a separate file labeled 'Revised Manuscript with Track Changes'.
- An unmarked version of your revised paper without tracked changes. You should upload this as a separate file labeled 'Manuscript'.

If you would like to make changes to your financial disclosure, please include your updated statement in your cover letter. Guidelines for resubmitting your figure files are available below the reviewer comments at the end of this letter.

If applicable, we recommend that you deposit your laboratory protocols in protocols.io to enhance the reproducibility of your results. Protocols.io assigns your protocol its own identifier (DOI) so that it can be cited independently in the future. For instructions see: <https://journals.plos.org/plosone/s/submission-guidelines#loc-laboratory-protocols>. Additionally, PLOS ONE offers an option for publishing peer-reviewed Lab Protocol articles, which describe protocols hosted on protocols.io. Read more information on sharing protocols at <https://plos.org/protocols?utm_medium=editorial-email&utm_source=authorletters&utm_campaign=protocols>.

We look forward to receiving your revised manuscript.

Kind regards,

Wen-Wei Sung, M.D., Ph.D.
Academic Editor
PLOS ONE

Journal Requirements:

When submitting your revision, we need you to address these additional requirements.

1. Please ensure that your manuscript meets PLOS ONE's style requirements, including those for file naming. The PLOS ONE style templates can be found at 
https://journals.plos.org/plosone/s/file?id=wjVg/PLOSOne_formatting_sample_main_body.pdf and 
https://journals.plos.org/plosone/s/file?id=ba62/PLOSOne_formatting_sample_title_authors_affiliations.pdf

Complied.

2. In your Data Availability statement, you have not specified where the minimal data set underlying the results described in your manuscript can be found. PLOS defines a study's minimal data set as the underlying data used to reach the conclusions drawn in the manuscript and any additional data required to replicate the reported study findings in their entirety. All PLOS journals require that the minimal data set be made fully available. For more information about our data policy, please see http://journals.plos.org/plosone/s/data-availability.

"Upon re-submitting your revised manuscript, please upload your study’s minimal underlying data set as either Supporting Information files or to a stable, public repository and include the relevant URLs, DOIs, or accession numbers within your revised cover letter. For a list of acceptable repositories, please see http://journals.plos.org/plosone/s/data-availability#loc-recommended-repositories. Any potentially identifying patient information must be fully anonymized.

Important: If there are ethical or legal restrictions to sharing your data publicly, please explain these restrictions in detail. Please see our guidelines for more information on what we consider unacceptable restrictions to publicly sharing data: http://journals.plos.org/plosone/s/data-availability#loc-unacceptable-data-access-restrictions. Note that it is not acceptable for the authors to be the sole named individuals responsible for ensuring data access.

We will update your Data Availability statement to reflect the information you provide in your cover letter.

The data used in the study are available as supplementary material to this article.

3. We note that Figure 1 in your submission contain [map/satellite] images which may be copyrighted. All PLOS content is published under the Creative Commons Attribution License (CC BY 4.0), which means that the manuscript, images, and Supporting Information files will be freely available online, and any third party is permitted to access, download, copy, distribute, and use these materials in any way, even commercially, with proper attribution. For these reasons, we cannot publish previously copyrighted maps or satellite images created using proprietary data, such as Google software (Google Maps, Street View, and Earth). For more information, see our copyright guidelines: http://journals.plos.org/plosone/s/licenses-and-copyright.
 
We require you to either (1) present written permission from the copyright holder to publish these figures specifically under the CC BY 4.0 license, or (2) remove the figures from your submission:
 
a. You may seek permission from the original copyright holder of Figure 1 to publish the content specifically under the CC BY 4.0 license.  
 
We recommend that you contact the original copyright holder with the Content Permission Form (http://journals.plos.org/plosone/s/file?id=7c09/content-permission-form.pdf) and the following text:
“I request permission for the open-access journal PLOS ONE to publish XXX under the Creative Commons Attribution License (CCAL) CC BY 4.0 (http://creativecommons.org/licenses/by/4.0/). Please be aware that this license allows unrestricted use and distribution, even commercially, by third parties. Please reply and provide explicit written permission to publish XXX under a CC BY license and complete the attached form.”
 
Please upload the completed Content Permission Form or other proof of granted permissions as an "Other" file with your submission.
 
In the figure caption of the copyrighted figure, please include the following text: “Reprinted from [ref] under a CC BY license, with permission from [name of publisher], original copyright [original copyright year].”
 
b. If you are unable to obtain permission from the original copyright holder to publish these figures under the CC BY 4.0 license or if the copyright holder’s requirements are incompatible with the CC BY 4.0 license, please either i) remove the figure or ii) supply a replacement figure that complies with the CC BY 4.0 license. Please check copyright information on all replacement figures and update the figure caption with source information. If applicable, please specify in the figure caption text when a figure is similar but not identical to the original image and is therefore for illustrative purposes only.
The following resources for replacing copyrighted map figures may be helpful:
 
USGS National Map Viewer (public domain): http://viewer.nationalmap.gov/viewer/
The Gateway to Astronaut Photography of Earth (public domain): http://eol.jsc.nasa.gov/sseop/clickmap/
Maps at the CIA (public domain): https://www.cia.gov/library/publications/the-world-factbook/index.html and https://www.cia.gov/library/publications/cia-maps-publications/index.html
NASA Earth Observatory (public domain): http://earthobservatory.nasa.gov/
Landsat: http://landsat.visibleearth.nasa.gov/
USGS EROS (Earth Resources Observatory and Science (EROS) Center) (public domain): http://eros.usgs.gov/#
Natural Earth (public domain): <http://www.naturalearthdata.com/>

Thank you very much for your comment. Figure 1, the map, is not copyrighted. It was developed based on the data obtained from a public domain (<http://www.naturalearthdata.com/>).

Additional Editor Comments:
This observational study provides a possible relationship between oral Polio vaccine and low incidence of COVID-19 infection. As there is no solid molecular evidence to support the relationship, more discussion and a list of limitations might be necessary for this revision.

Thank you very much for your comment. Please note that the real molecular data are rarely available for causal relationships in such diseases, including todays SARS-CoV-2 and CoVID-19. Some (like HIV) are an exception. However, if by this you mean a reasonable and known molecular mechanism of a likely explanation, it would certainly be by activating TLR and related receptors reacting to PAMP signals and thereby activating IFN-Type 1 genes, which in turn affect various immediate antiviral pathways. NK cells are also activated. However, we certainly agree we have not shown that here. We have already mentioned limitations of the study. In the revised version, we clearly show the Limitations section. We also added the probable protective effect of other live attenuated vaccines such as BCG and the probable role of cross-reacting antibodies on the results, as other limitations of the study.

Reviewers' comments:

Reviewer's Responses to Questions

**Comments to the Author**

1. Is the manuscript technically sound, and do the data support the conclusions?

The manuscript must describe a technically sound piece of scientific research with data that supports the conclusions. Experiments must have been conducted rigorously, with appropriate controls, replication, and sample sizes. The conclusions must be drawn appropriately based on the data presented.

Reviewer #1: Yes

Reviewer #2: Partly

2. Has the statistical analysis been performed appropriately and rigorously?

Reviewer #1: Yes

Reviewer #2: Yes

3. Have the authors made all data underlying the findings in their manuscript fully available?

The [PLOS Data policy](http://www.plosone.org/static/policies.action#sharing) requires authors to make all data underlying the findings described in their manuscript fully available without restriction, with rare exception (please refer to the Data Availability Statement in the manuscript PDF file). The data should be provided as part of the manuscript or its supporting information, or deposited to a public repository. For example, in addition to summary statistics, the data points behind means, medians and variance measures should be available. If there are restrictions on publicly sharing data—e.g. participant privacy or use of data from a third party—those must be specified.

Reviewer #1: Yes

Reviewer #2: Yes

4. Is the manuscript presented in an intelligible fashion and written in standard English?

PLOS ONE does not copyedit accepted manuscripts, so the language in submitted articles must be clear, correct, and unambiguous. Any typographical or grammatical errors should be corrected at revision, so please note any specific errors here.

Reviewer #1: Yes

Reviewer #2: Yes

5. Review Comments to the Author

Please use the space provided to explain your answers to the questions above. You may also include additional comments for the author, including concerns about dual publication, research ethics, or publication ethics. (Please upload your review as an attachment if it exceeds 20,000 characters)

Reviewer #1: The authors performed a population study utilizing publicly available data on the number of COVID cases per country and their usage of oral poliovirus vaccine vs inactivated poliovirus vaccine, as well as their human development index. The authors' data support their conclusion, but there are many variables that would go into the spread of the virus during the pandemic beyond HDI, GDP, and overall health care.

The authors should add in population density as another variable to assess.

Thank you very much for your comment. We added the population density to our study. It was not significantly different (p=0.201) in countries using OPV vs IPV (Table 1). Nevertheless, we’ve also added it to the model but it did not change anything significantly; it was not a significant predictor in the model. Therefore, we decided not to change the model.

The authors need to fix figure 3 which is cutoff.

We appreciate this suggestion and have complied by making this change to Figure 3.

The authors should include additional discussion on the potential roles that lockdowns and international travel could have played in the observed differences.

Thanks for raising this important point. The effects of lockdowns and international travel bans were hard to account for in our study since policies changed with time. However, we added the mean stringency index, an index reflecting the level of restrictions imposed in a given country, to our study. The mean was not significantly different between countries using OPV vs those using IPV. We have now added this in detail in the methodology section, Table 1, and Discussion.

The authors should specifically state whether the outliers shown in figure 2 were included in the analyses or excluded. If they were excluded, they should provide the statistical criteria for the exclusion, and provide a secondary graph with them included.

Thank you very much for making this suggestion. All the outliers were included in the analyses.

Reviewer #2: The overall observation of this study might be true but the role of OPV is more hypothetical. What the authors failed to conclude with sufficient evidence is whether the observed protection for SARS-CoV-2 in countries with OPV is really due to OPV, BCG (widely used in LMICs) or many other rampant viral and bacterial infection with high frequency. Its well documented that non specific innate immunity provides protection to some degree from several other pathogens. Not only innate immunity, recent experimental evidences suggest that antibodies generated (humorl response) in response to other viral pathogens such as HIV and Flu cross reacts with SARS-COV-2 and might provide protection indirectly through antibody dependent virus clearance rather than direct neutralization. To conclude the specific role of OPV, a more direct comparison need to be done within the same environmental setting by comparing the number of COVID-19 infection among the population with OPV and without OPV vaccination. The circulating OPV strain among the population through improper hygiene in LMICs is not expected to provide significant dosing for indirect protection against SARS-CoV-2.

Thank you very much. The reviewer has raised an important point. We agree this is in fact a limitation of this study, mainly due to the nature of the ecological studies. We addressed this point in the Limitations section of the Discussion.

6. PLOS authors have the option to publish the peer review history of their article If published, this will include your full peer review and any attached files.


If you choose “no”, your identity will remain anonymous but your review may still be made public.


**Do you want your identity to be public for this peer review?** For information about this choice, including consent withdrawal, please see our [Privacy Policy](https://www.plos.org/privacy-policy).

Reviewer #1: No

Reviewer #2: No

[NOTE: If reviewer comments were submitted as an attachment file, they will be attached to this email and accessible via the submission site. Please log into your account, locate the manuscript record, and check for the action link "View Attachments". If this link does not appear, there are no attachment files.]

While revising your submission, please upload your figure files to the Preflight Analysis and Conversion Engine (PACE) digital diagnostic tool, <https://pacev2.apexcovantage.com/>. PACE helps ensure that figures meet PLOS requirements. To use PACE, you must first register as a user. Registration is free. Then, login and navigate to the UPLOAD tab, where you will find detailed instructions on how to use the tool. If you encounter any issues or have any questions when using PACE, please email PLOS at [figures@plos.org](mailto:figures@plos.org). Please note that Supporting Information files do not need this step.

*In compliance with data protection regulations, you may request that we remove your personal registration details at any time.* [*(Remove my information/details)*](https://www.editorialmanager.com/pone/login.asp?a=r)*. Please contact the publication office if you have any questions.*
